# Decolonizing global health governance through knowledge: a perspective on epistemic governance

**DOI:** 10.3389/fpubh.2026.1956038

**Published:** 2026-09-24

**Authors:** Abeer Ismail, Aya Abu Aqab, Dalal Iriqat

**Affiliations:** 1 Department of Public Health, Faculty of Graduate Studies, Al-Quds University, Gaza, Palestine; 2 Department of Public Health, Faculty of Graduate Studies, Al-Quds University, Jerusalem, Palestine; 3 Department of Social Sciences, Faculty of Graduate Studies, Arab American University AAUP, Jenin, Palestine

**Keywords:** decolonization, epistemic justice, global health governance, health data sovereignty, knowledge co-production, technology transfer

## 1 Introduction

Global health has become a major focus of decolonization debates, with scholarship documenting persistent colonial structures in funding, governance, leadership, and institutional practices while proposing pathways for reform (1–3). However, less attention has been paid to the epistemic foundations of global health governance specifically, what constitutes valid knowledge, who is authorized to produce it, and whose expertise is considered universally applicable. This article argues that global health governance is fundamentally a knowledge practice, with colonial legacies embedded in the epistemic systems that shape research authorship, guideline development, and technology transfer. Drawing on decolonial theory, the decolonizing global health literature, and STS (4–6), it contends that decolonizing global health requires transforming not only institutional representation but also the knowledge structures that define health problems, generate evidence, and guide global responses. The article develops the concept of epistemic governance, illustrates it through global health authorship asymmetries and the WHO mRNA vaccine technology transfer programme in South Africa, and concludes by outlining mechanisms, challenges, and future directions for decolonizing knowledge in global health governance.

This article is presented as a perspective on epistemic governance in global health rather than as an empirical study, systematic review, or scoping review. It draws on existing scholarship and critical reflections to examine how knowledge production, validation, and circulation shape power relations within global health governance. The aim is to advance a conceptual and critical argument for decolonizing global health governance through greater recognition of diverse knowledge systems, epistemic pluralism, and more equitable structures of knowledge production and decision-making.

## 2 Literature positioning

### 2.1 Decolonial theory and the coloniality of knowledge

Decolonial theory argues that colonialism persists through enduring structures of power and knowledge rather than ending with political independence. Quijano's concept of the coloniality of power explains how colonial hierarchies continue to shape knowledge production, including the privileging of Eurocentric standards in global health (4). Mignolo further contends that decolonization requires delinking from the assumption that Northern biomedical epistemology is the universal standard for evaluating health knowledge (5). Similarly, Wyse argues that this dominance results in epistemicide, whereby alternative forms of knowledge are marginalized through hierarchies of evidentiary legitimacy that privilege scientific and institutional evidence over local, community, and context-specific knowledge (7).

### 2.2 The decolonizing global health literature

A growing body of literature has documented the persistence of colonial structures in global health institutions, including inequities in funding, research partnerships, workforce composition, and governance, while calling for structural reform (1–3). Abimbola's work provides a key foundation for the concept of epistemic governance by highlighting systematic inequalities in global health knowledge production. He demonstrates that researchers from high-income countries often dominate first and senior authorship in studies conducted in low- and middle-income countries (LMICs) despite substantial local contributions (6, 8). He also argues that global health institutions privilege knowledge generated by those who produce and generalize theory over the context-specific expertise of practitioners and advocates, reinforcing epistemic hierarchies and limiting the recognition of locally grounded knowledge (1).

### 2.3 Science and technology studies and Indigenous data sovereignty

Jasanoff's concept of co-production argues that scientific knowledge and governance are mutually constituted, meaning that global health tools, guidelines, and technologies simultaneously shape knowledge and authority by defining whose expertise is considered legitimate (9). Similarly, Smith's work on decolonizing methodologies highlights how research practices have historically reinforced colonial control and advocates for community-led and reciprocal approaches to knowledge production (10). These principles are reflected in the Ownership, Control, Access, and Possession (OCAP) framework, which emphasizes Indigenous ownership and control of health data (11), and the Collective Benefit, Authority to Control, Responsibility, and Ethics (CARE) Principles, which promote Indigenous data governance based on collective benefit, authority, responsibility, and ethics alongside conventional data-sharing practices (12).

### 2.4 The gap

While the decolonizing global health literature has documented colonial inequities in authorship, funding, and institutional leadership, it has paid less attention to the underlying epistemic structures that sustain these patterns. Conversely, decolonial theory and STS provide conceptual tools for understanding knowledge hierarchies but have rarely been applied to the institutional mechanisms of global health governance. This article bridges these perspectives through the concept of epistemic governance, linking empirical evidence of global health inequities with a deeper analysis of the epistemological foundations that reproduce them.

## 3 Conceptual framework: epistemic governance of global health

This section develops four linked concepts, adapted specifically to global health governance: epistemic governance, knowledge regimes of global health, epistemic exclusion, and epistemic pluralism/co-production.

### 3.1 Epistemic governance

Epistemic governance refers to the systems, institutions, and practices that determine what constitutes valid knowledge in global health and who has the authority to define health problems and solutions. It operates through mechanisms such as research authorship, peer review, WHO guideline development, disease metrics, and technology-transfer processes. These epistemic structures shape how health problems are recognized and addressed, reflecting underlying assumptions about whose knowledge is considered legitimate rather than functioning as neutral technical processes.

### 3.2 Knowledge regimes of global health

A global health knowledge regime comprises the institutions, funders, academic centers, and journals that shape the production and circulation of global health knowledge. Rather than resulting from deliberate coordination, these regimes are sustained by funding, publication, and credentialing systems that privilege standardized, measurable, and generalizable evidence over context-specific local expertise. Consequently, they systematically favor the knowledge of academics and policy experts over that of practitioners and communities with direct experience of local health systems (6).

### 3.3 Epistemic exclusion

Epistemic exclusion in global health refers to the systematic marginalization of context-specific, practice-based, and community knowledge from what is recognized as legitimate evidence. This is reflected in authorship practices that prioritize foreign-led research teams over local researchers and health workers in LMICs, despite their essential contributions (7). Such exclusion is reinforced by publication norms, funding systems, and academic incentives that structurally privilege externally generated knowledge.

### 3.4 Epistemic pluralism and co-production

Epistemic pluralism refers to global health governance that recognizes multiple knowledge systems and producers as equally legitimate rather than hierarchically ranked (9). In practice, this requires treating LMIC researchers, health workers, and communities as equal partners in designing research, developing technical protocols, and defining evidence standards, rather than merely as data providers or implementers. However, as illustrated in Section 4.2, formal commitments to co-production do not necessarily result in meaningful epistemic inclusion.

## 4 Case illustrations

### 4.1 Authorship asymmetries as structural epistemic exclusion

Abimbola's analysis shows that global health research on LMICs is disproportionately led by authors from high-income countries, despite relying heavily on the expertise and contributions of LMIC researchers and health workers-a phenomenon described as the “foreign gaze” (6, 7). Beyond professional recognition, authorship serves as a mechanism of epistemic governance, conferring intellectual authority, career advancement, and influence over guideline development and policy-making. As a result, inequities in authorship reinforce broader patterns of epistemic exclusion within global health institutions. Abimbola further argues that global health systems privilege the theoretical and systems-oriented knowledge of “professors” and “engineers” over the practical and justice-oriented knowledge of “plumbers” and “emancipators,” thereby sustaining knowledge hierarchies through the field's own evaluative standards rather than explicit discrimination (6, 7).

### 4.2 The WHO mRNA technology transfer programme: co-production under contested conditions

The WHO-Medicines Patent Pool mRNA vaccine technology transfer programme, established in 2021 in response to global COVID-19 vaccine inequities, aimed to strengthen LMIC manufacturing through a South African hub led by Afrigen Biologics and Vaccines and partner institutions (13–16). Although the initiative expanded technology sharing and supported vaccine production capacity across multiple countries, evidence suggests that governance and decision-making authority remained largely centralized within WHO and MPP. As a result, while technical knowledge was transferred, Southern partners had limited influence over priorities, commercialization, and independent decision-making (13). This case highlights the distinction between technology transfer and epistemic co-production, demonstrating that genuine decolonization requires not only sharing technical expertise but also redistributing authority over knowledge production, governance, and health priorities (13–16).

## 5 Mechanisms for decolonizing knowledge in global health governance

Building on the contrast above, this section outlines four mechanisms through which global health governance might move from epistemic exclusion toward genuine epistemic pluralism.

### 5.1 Equitable authorship and research partnership standards

Journals, funders, and universities can promote more equitable global health research by adopting authorship policies that recognize contextual expertise and local leadership alongside traditional academic contributions. Measures such as mandatory local co-authorship, revised promotion criteria that encourage equitable authorship, and more diverse editorial boards can help reduce authorship inequities. However, as Abimbola cautions, these reforms must address the underlying “foreign gaze” rather than becoming symbolic solutions that leave deeper epistemic inequalities unchanged (7).

### 5.2 Substantive rather than technical-only technology transfer

The mRNA technology transfer case demonstrates that decolonizing global health requires transferring not only technical knowledge but also decision-making authority over its prioritization, commercialization, and implementation. Achieving this demands stronger commitments to affordability, public health priorities, and greater autonomy for Southern manufacturing hubs within technology-transfer programmes (13).

### 5.3 Indigenous and community health data sovereignty

The OCAP principles (10) and the CARE Principles for Indigenous Data Governance (12) provide practical frameworks for ensuring community authority over health data. Applied to global health governance, these principles support giving LMIC institutions and communities a formal role in governing how health data are collected, shared, integrated into global metrics, and used to guide research, funding, and policy decisions (10, 12).

### 5.4 Plural evidentiary standards in guideline development and knowledge synthesis

The WHO guideline-development processes and global health evidence synthesis could be strengthened by formally recognizing practice-based and context-specific knowledge alongside traditional sources such as randomized trials and large-scale surveys. This would elevate the contributions of Abimbola's (7) “plumbers” and “emancipators” and require reforming existing evidentiary hierarchies, including frameworks used to evaluate evidence for global health recommendations.

### 5.5 Building LMIC-led knowledge infrastructures

LMICs and their researchers have an important role to play in reshaping the global health knowledge ecosystem. Rather than remaining primarily producers of data for externally defined research agendas, LMIC researchers, universities, professional associations, and public agencies can establish, strengthen, and sustain knowledge infrastructures that are owned, governed, and operated by LMIC institutions. These may include scholarly journals, research consortia, professional societies, conferences, repositories, and policy platforms that enable LMIC researchers and practitioners to define research priorities, generate and disseminate locally relevant evidence, and influence global health agendas. Such initiatives require investment in local editorial and peer-review capacity, open-access publishing, research communication, and regional knowledge networks. Equally important, universities, funders, and promotion committees should ensure that LMIC researchers receive full recognition for the intellectual contributions of their work regardless of where it is published. Publication in LMIC-led or regionally governed journals should not be regarded as evidence of lower scholarly quality or capacity. Academic assessment should therefore place greater emphasis on the quality, originality, methodological rigor, relevance, leadership, and societal contribution of research rather than treating journal prestige, citation-based metrics, or impact factor as proxies for intellectual capacity. Strengthening LMIC-led publishing platforms can thus create legitimate and sustainable spaces for locally grounded scholarship while improving the visibility and dissemination of research produced in these settings. The goal should not be to create isolated parallel knowledge systems, but to strengthen autonomous LMIC-led infrastructures that participate actively in international knowledge exchange while retaining meaningful local ownership, editorial independence, and decision-making authority. International funders and organizations can support this process through sustained financing, equitable partnerships, editorial capacity strengthening, open-access dissemination, and regional research networks. Such investments can help shift LMIC researchers from being primarily data producers and implementers to becoming knowledge producers, research leaders, agenda-setters, editors, reviewers, and institutional stewards of global health knowledge.

## 6 Tensions and limits

Three key objections require consideration. First, valuing contextual and practice-based knowledge does not mean rejecting rigorous scientific evidence; rather, it challenges the assumption that a single evidentiary model should dominate and argues for recognizing diverse forms of valid knowledge while maintaining standards of reliability and effectiveness. Second, although the WHO mRNA technology transfer programme represents meaningful progress in technical knowledge-sharing, it demonstrates that formal co-production arrangements do not necessarily transform underlying decision-making and epistemic hierarchies (13). Third, authorship reform is not merely an issue of academic recognition; because authorship influences access to expert networks, advisory roles, and policy-making spaces, inequities in authorship can shape whose knowledge informs global health governance (7).

## 7 Conclusion

This article argues that decolonizing global health governance requires addressing the knowledge architectures through which health problems are defined, measured, and addressed, not only institutional structures. By integrating decolonial theory, evidence on global health inequities, and STS, it develops the concept of epistemic governance to explain how rship practices, guideline processes, and technology-transfer arrangements reproduce knowledge hierarchies despite formal reforms. The cases of global health authorship inequalities and the WHO mRNA technology transfer programme demonstrate the persistence of epistemic exclusion and the challenges of achieving genuine knowledge co-production. Decolonizing global health therefore requires shifting not only technical knowledge but also epistemic and decision-making authority. Future research should further examine technology-transfer models, research partnerships, and community-led data governance approaches as pathways toward more equitable global health knowledge systems.
